# Gravitational distribution of regional opening and closing pressures, hysteresis and atelectrauma in ARDS evaluated by electrical impedance tomography

**DOI:** 10.1186/s13054-020-03335-1

**Published:** 2020-10-22

**Authors:** Gaetano Scaramuzzo, Elena Spinelli, Savino Spadaro, Alessandro Santini, Donatella Tortolani, Francesca Dalla Corte, Antonio Pesenti, Carlo Alberto Volta, Giacomo Grasselli, Tommaso Mauri

**Affiliations:** 1 Department of Morphology, Surgery and Experimental Medicine, Intensive Care Unit, Azienda Ospedaliera Universitaria Sant’Anna Hospital, Ferrara, Italy; 2 Department of Anesthesia, Critical Care and Emergency, Fondazione IRCCS Ca’ Granda Ospedale Maggiore Policlinico, Via F. Sforza 35, 20122 Milan, Italy; 3 Department of Anaesthesia and Intensive Care Medicine, Humanitas Clinical and Research Centre-IRCCS, Rozzano, Milan Italy; 4 Department of Pathophysiology and Transplant, University of Milan, Milan, Italy

**Keywords:** Electrical impedance tomography, Hysteresis, VILI, ARDS

## Abstract

**Background:**

The physiological behavior of lungs affected by the acute respiratory distress syndrome (ARDS) differs between inspiration and expiration and presents heterogeneous gravity-dependent distribution. This phenomenon, highlighted by the different distribution of opening/closing pressure and by the hysteresis of the pressure–volume curve, can be studied by CT scan, but the technique expose the patient to radiations, cannot track changes during time and is not feasible at the bedside. Electrical impedance tomography (EIT) could help in assessing at the bedside regional inspiratory and expiratory mechanical properties. We evaluated regional opening/closing pressures, hysteresis and atelectrauma during inspiratory and expiratory low-flow pressure–volume curves in ARDS using electrical impedance tomography.

**Methods:**

Pixel-level inspiratory and expiratory PV curves (PV_pixel_) between 5 and 40 cmH_2_O were constructed integrating EIT images and airway opening pressure signal from 8 ARDS patients. The lower inflection point in the inspiratory and expiratory PV_pixel_ were used to find opening (OP_pixel_) and closing (CP_pixel_) pressures. A novel atelectrauma index (AtI) was calculated as the percentage of pixels opening during the inspiratory and closing during the expiratory PV curves. The maximal hysteresis (HysMax) was calculated as the maximal difference between normalized expiratory and inspiratory PV curves. Analyses were conducted in the global, dependent and non-dependent lung regions.

**Results:**

Gaussian distribution was confirmed for both global OP_pixel_ (r^2^ = 0.90) and global CP_pixel_ (r^2^ = 0.94). The two distributions were significantly different with higher values for OP_pixel_ (p < 0.0001). Regional OP_pixel_ and CP_pixel_ distributions were Gaussian, and in the dependent lung regions, both were significantly higher than in the non-dependent ones (p < 0.001). Both AtI and the HysMax were significantly higher in the dependent regions compared to the non-dependent ones (p < 0.05 for both).

**Conclusions:**

Gravity impacts the regional distribution of opening and closing pressure, hysteresis and atelectrauma, with higher values in the dorsal lung. Regional differences between inspiratory and expiratory lung physiology are detectable at the bedside using EIT and could allow in-depth characterization of ARDS phenotypes and guide personalized ventilation settings.

**Graphic abstract:**

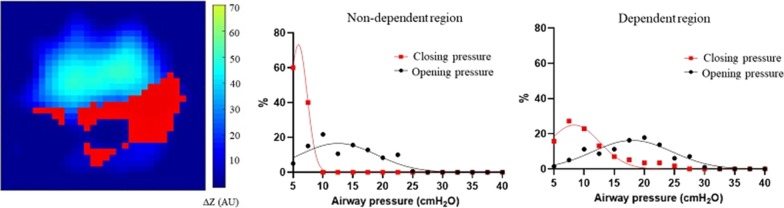

## Background

Acute respiratory distress syndrome (ARDS) is characterized by the development of bilateral acute lung inflammation and edema as a consequence of direct or indirect injury [1]. Although inflammation is diffuse, lung edema is not homogeneously distributed and its impact on the mechanical properties of each lung region depends on several factors, such as gravity [2] or distance from the pleural surface [3]. The alveolar distending pressure, i.e., the transpulmonary pressure, presents, therefore, an uneven distribution in the lungs. When transpulmonary pressure (P_L_) becomes negative, terminal airways and alveoli tends to collapse. Given the regional heterogeneity of P_L_, the threshold at which each lung unit opens and closes (i.e., regional P_L_ = 0 cmH_2_O) does not correspond to a univocal pressure measured at airway opening [4]. Previous studies hypothesized [5] and measured [6] how: (1) recruitment and derecruitment are distributed along the entire pressure–pressure–volume curve and depend from gravity, (2) opening pressures are higher than closing pressure and (3) every patient has unique distribution of opening and closing pressures [6]. Temporal heterogeneity of ARDS can further increase the variety of these physiological mechanisms.

The pressure–volume curve (PV) of the respiratory system has been used in the last decades to describe the effect of increasing and decreasing pressure in the respiratory system in a quasi-static condition. Its shape during inspiration and expiration is different, since the pressure needed to inflate is higher than the one at which collapse happens (hysteresis), probably for the dynamic action of the surfactant layer and its role in reducing superficial tension [7]: Indeed, hysteresis is absent when water is used to expand isolated animal lungs [8]. The lower inflection point (LIP) of the inspiratory PV curve indicates the pressure for terminal airways or alveoli to open, while the LIP of the expiratory limb represents the closing pressure. Despite its sound physiological basis, the use of the PV curve built from pressure and volume measured at airway opening expresses only the average behavior of the lung and it is not able to highlight the heterogeneity of regional lung mechanics [9].

Knowing the patient’s regional distribution of opening and closing pressures can help in treating physicians to set mechanical ventilation and potentially improve the comprehension of regional pathophysiology and therefore lung protection. It is well known how the cyclical opening and closing, also called atelectrauma [10, 11], can amplify the inflammatory reaction in ARDS, being one of the main determinants of ventilator-induced lung injury (VILI). Similarly, regional hysteresis could represent a simplified method to assess potential for lung recruitment at the bedside. So far, lung CT scan was used to highlight the distribution of opening and closing pressure, but this technique is not feasible at the bedside and exposes the patients to ionizing radiations [6].

Electrical impedance tomography is a radiation-free technique which has been increasingly used in the last decade to monitor ventilation [12–15]. Regional inspiratory pressure–volume curves have been previously built in ARDS patients, by integrating pressure signals and EIT images [9]. Theoretically, the integration of EIT and airway pressure signal would allow to generate the inspiratory and expiratory pressure–volume curves of different functional lung units at the bedside and determine the regional behavior of opening and closing pressures, of the magnitude of hysteresis and, finally, of the risk of atelectrauma. Consequently, our hypothesis was that we would be able to detect regional opening/closing pressures using electrical impedance tomography and evaluate their gravity-dependent regional distribution in patients affected by ARDS.

## Methods

### Study population

Patients affected by ARDS [1], aged ≥ 18 years, sedated and paralyzed as per clinical decision were enrolled. Exclusion criteria were: refusal to participate to the study, pregnancy, unstable hemodynamics, pneumothorax, severe chronic obstructive pulmonary disease, impossibility to correctly position the EIT belt (e.g., chest drainage, surgical wound dressings) and contraindications to EIT monitoring (e.g., pacemaker, automatic implantable cardioverter defibrillator). The ethical committee of Milan Policlinico Hospital (reference number 364_2017) approved the study, and informed consent was obtained following local regulations. At enrollment, we collected demographic and clinical data of each patient.

### Patients’ monitoring and PV curves

All patients were in the supine semi-recumbent position. Pressure at airway opening (Pao) and flow (f) were recorded and processed by a dedicated data acquisition system (Colligo System, Elekton, Milan, Italy). Volume was calculated as flow integral. A 16-electrode silicon EIT belt was placed around the chest and connected to a dedicated monitor (PulmoVista® 500, Dräger, Lübeck, Germany). All patients underwent a low-flow inflation/deflation maneuver using the built-in ventilator tool (HAMILTON-S1, Hamilton Medical AG, Bonaduz, Switzerland) starting from Pao = 5 cmH_2_O to Pao = 40 cmH_2_O and back to Pao = 5 cmH_2_O as previously described [9, 16]. Driving pressure (DP) was calculated as the difference between end-inspiratory pressure (plateau pressure) and end-expiratory pressure after 2-s holds before performing the PV curves at PEEP = 5 cmH_2_O and with tidal volume = 6–8 ml/kg/IBW.

### Pixel-level PV curves

Airway pressure waveform and EIT images during each maneuver were synchronized offline at intervals of Pao of 2.5 cmH_2_O. The variation of impedance (ΔZ) in each pixel during the maneuver was used with the corresponding ΔPao to build pixel-level PV curves (PV_pixel_) for the inspiratory (PV_pixel-I_) and expiratory (PV_pixel-E_) maneuver. Each PV_pixel-I_ and PV_pixel-E_ curve was fitted in the equation of a sigmoid [17], and fitting was considered effective if r^2^ > 0.9. The PV curves with a r^2^ < 0.9 were discarded (poor fitting). For each inspiratory and expiratory PV_pixel_ curve, the lower inflection point (LIP_pixel_) was mathematically identified [9]. The Pao corresponding to the inspiratory LIP_pixel_ was considered as opening pressure for that unit (OP_pixel_), while the pressure corresponds to the expiratory LIP_pixel_ as closing pressure (CP_pixel_). If the PV_pixel_ curve was devoid of LIP_pixel_, OP_pixel_ or CP_pixel_ was likely below the starting pressure for the PV maneuver (5 cmH_2_O). The distribution of OP_pixel_ and CP_pixel_ for each patient was fitted into a Gaussian equation, as previously described by Crotti et al. [6]. Then, the curve for OP_pixel_ and CP_pixel_ for all patients was created using the cumulative mean ± SEM at each pressure interval (2.5 cmH_2_O starting from PEEP 5 cmH_2_O). The distribution was expressed as percentage of the total pixels with valid LIP_pixel_, defined as:

$${\text{Valid}}\;{\text{LIP}}_{{{\text{pixel}}}} \; = \;{\text{ventilated}}\;{\text{pixels - (poor}}\;{\text{fitting}}\;{\text{pixels}}\;{\text{ + }}\;{\text{no}}\;{\text{LIP}}\;{\text{ pixels)}}$$

The distribution of opening/closing pressures was evaluated in the whole lung and in the dependent (ROI_D_) and non-dependent (ROI_ND_) regions of interest. Classically, the dependent and non-dependent regions are constructed by dividing the matrix of the EIT image into two different regions based on a fixed threshold. Since the position of the lungs can be variable, we decided to adopt a more precise method, finding the centroid of each cumulative image (entire ventilated lung) which represents the mean position of all the points in all of the coordinate directions. The two regions, the patient’s dorsal (ROI_D_) and ventral (ROI_ND_) lung region, were therefore defined by being, respectively, below and above the calculated centroid of each cumulative EIT image. Pixels ventilated less than 10% of the maximal pixel’s ∆Z were excluded from the EIT analysis.

### Atelectrauma index

The atelectrauma index (AtI) was calculated for each patient as the percentage of pixels with both LIP_pixel-I_ and LIP_pixel-E_ values between 5 and 40 cmH_2_O (defined as pixels opening and closing in the following formula), and therefore, that fulfilled both the following:

- Presence of OP_pixel_ between 5 and 40 cmH_2_O
- Presence of CP_pixel_ between 5 and 40 cmH_2_O

divided by the total number of pixels receiving ventilation (Valid LIP_pixel_ + poor fitting pixels + No LIP pixels) during the inspiratory maneuver:

$${\text{AtI}} = \frac{{{\text{pixels}}\;{\text{opening}}\;{\text{and}}\;{\text{closing}}}}{{{\text{ventilated}}\;{\text{pixels}}}}*100$$

The AtI was calculated for the global lung and for the dependent (AtI_D_) and non-dependent (AtI_ND_) region as the number of pixels opening during PVinsp and closing during PVexp in the global lung or in each ROI divided by the total number of pixels increasing aeration during PVinsp in the whole lung or in each ROI, respectively.

### Hysteresis

The normalized maximal hysteresis (HysMAX) was calculated as the maximal difference between the expiratory and inspiratory limb of the PV curve after normalizing the variation of volume of each curve, derived from the EIT signal, between 0 and 1 (see Additional file 1: online data supplement, figure S4). The pressure corresponding to HysMAX was assessed in each patient and termed Pao_HysMAX_. The calculation of HysMAX and Pao_HysMAX_ was performed using the EIT signal for the global lung and for the dependent and non-dependent lung regions (Additional file 1: figure S4, *online supplement)*. EIT data analysis was performed using MATLAB R2018b (The MathWorks, Inc., Natick, Massachusetts, USA) and GraphPad prism (GraphPad Software, La Jolla California USA, www.graphpad.com).

### Statistical analysis

Sample size was similar to previous physiological studies on ARDS patients [6, 18]. Data are expressed as mean ± SD, mean ± SEM and median [IQR]. The cumulative OP_pixel_ and CP_pixel_ curves in the global lung and in each ROI were tested to evaluate if they could belong to the same distribution using the extra sum of squares F test. Moreover, the extra sum of squares F test was used to compare the distribution of dependent vs non-dependent OP_pixel_ and CP_pixel_. Spearman's rank-order correlation was used to test correlation between ranked variables. Wilcoxon signed-rank test was used to test differences among related samples. For all tests, a p value < 0.05 was considered significant. Statistical analysis was performed using GraphPad Prism version 8.3.0 for Windows (GraphPad Software, La Jolla California USA, www.graphpad.com).

## Results

### Patients’ characteristics

We analyzed data from 8 patients with mild and moderate ARDS, 4 males and 4 females, aged 68 (63–75) years with a median BMI of 27 (26–28) kg/m^2^ and a PaO_2_/FiO_2_ of 208 (185–237). Patients’ characteristics at enrollment are reported in Table 1.

**Table 1:** Patients’ main characteristics at enrollment on clinical settings

Gender (M:F)	4:4
Age (years)	68 [63–75]
BMI (Kg/m^2^)	27 [26–28]
SAPS II at ICU admission	56 [53–71]
Days of intubation before study	3 [2–5]
PaO_2_/FiO_2_	208 [185–237]
PaCO_2_ (mmHg)	37 [37–40]
pH	7.42 [7.37–7.45]
FiO_2_ (%)	45 [41–49]
PEEP (cmH_2_O)	12 [10–14]
Driving pressure (cmH_2_O)^a^	8.5 [7.7–9.0]
Respiratory system compliance (ml/cmH_2_O) ^a^	46 [42–59]
Outcome: survivors	6/8

Data expressed as median [IQR]

*BMI* body mass index, *SAPSII* simplified acute physiology score II, *ICU* intensive care unit, *PaO*_*2*_*/FiO*_*2*_ partial pressure of arterial oxygen on inspired fraction of oxygen ratio, *PEEP* positive end-expiratory pressure

^a^Measured at PEEP = 5cmH_2_O

### Opening and closing pressure

**Fig. 1:**
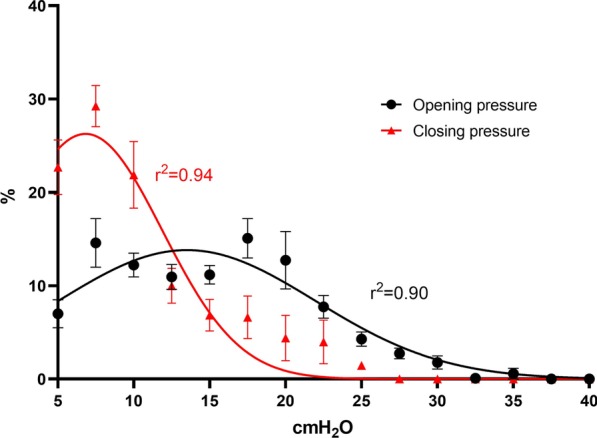
EIT-derived Distribution of opening/closing pressures. Distribution of EIT-derived opening and closing pressures in the global lung parenchyma. Mean ± SEM of 8 patients, Gaussian distribution, extra sum of fit, F-Test

**Fig. 2:**
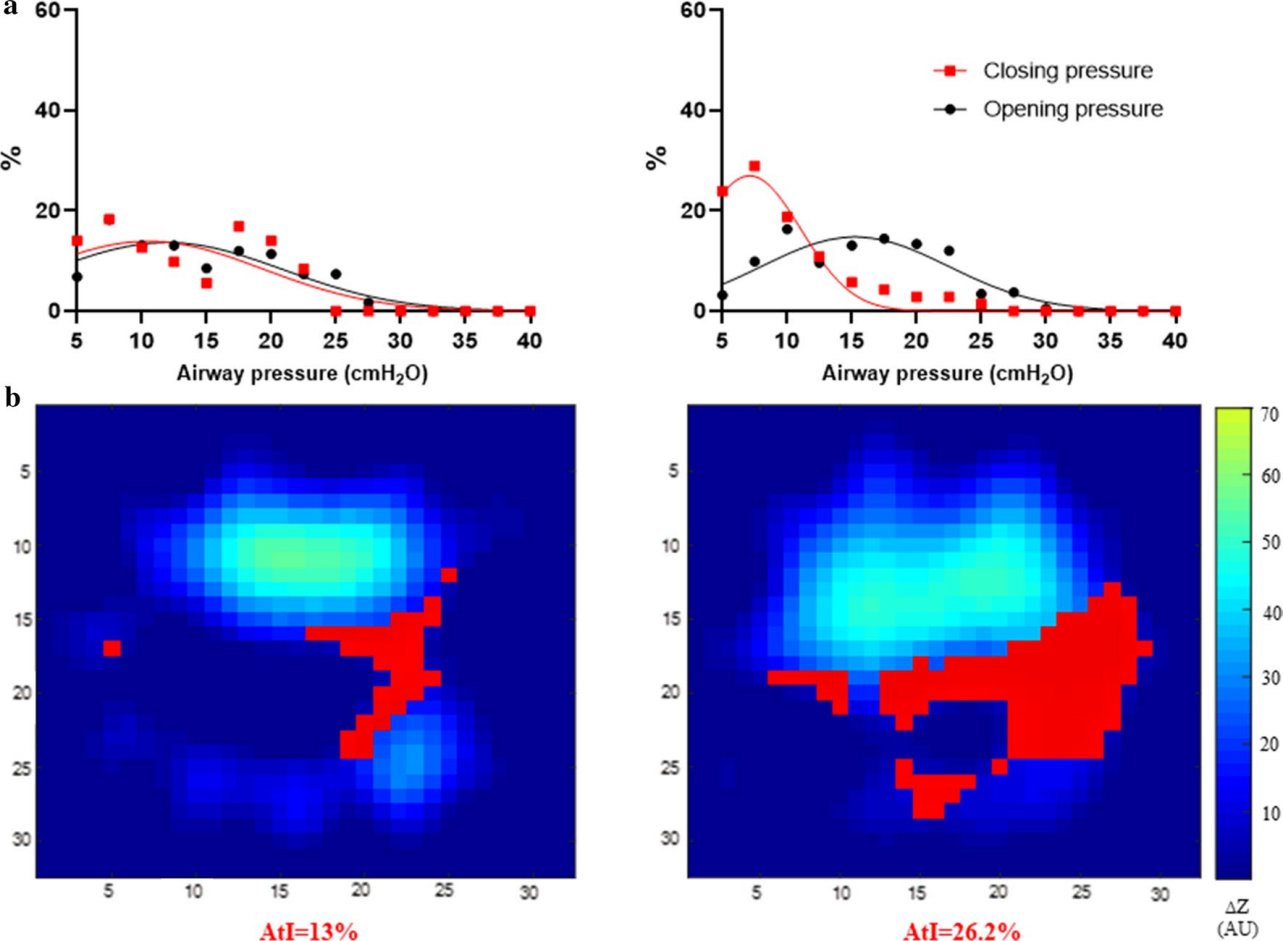
Example of regional opening/closing pressures (**a**) and atelectrauma index curves (**b**) in two representative patients. Distribution of opening/closing pressure in two representative patients (**a**) and the corresponding representation of atelectrauma index (**b**). Red pixels: pixels with inspiratory regional LIP along the inspiratory limb of the PV curve and expiratory regional LIP along the expiratory limb of the PV curve, DZ = relative change of pixel impedance. Images of tidal change during the PV maneuver, pixels ventilated > 10% of the max pixel are displayed. AtI = atelectrauma index (percentage of opening/closing pixels on total ventilated pixels)

By analyzing data from 8 inspiratory and 8 expiratory low-flow PV maneuvers, we obtained in total 3629 PV_pixel-I_ and 3657 PV_pixel-E_. Of these, 56.5% PV_pixel-I_ and 38.8% PV_pixel-E_ presented LIP_pixel_, while 11 PV_pixel-I_ and 7 PV_pixel-E_ were discarded for poor fitting (r^2^ < 0.9, Additional file 1: table S3). When analyzing opening and closing pressures distribution from EIT-derived normalized pixel-level pressure–volume curves in the whole lung, the normal Gaussian distribution fitted well both for OP_pixel_ (global mean value 13.5 ± 8.0 cmH_2_O, r^2^ = 0.9) and for CP_pixel_ (global mean value 6.8 ± 5.1 cmH_2_O, r^2^ = 0.94, Fig. 1). We confirmed that the two Gaussian distributions were significantly different, with higher values for OP_pixel_, since a simpler Gaussian fitting model for OP_pixel_ and CP_pixel_ could not improve goodness of fit (p < 0.0001, Additional file 1: table S5). However, patient-level distribution showed large variability, suggesting that average values should be interpreted cautiously and that these measures should be individualized (Fig. 2, Additional file 1: S2).

### Regional opening and closing pressure

**Fig. 3:**
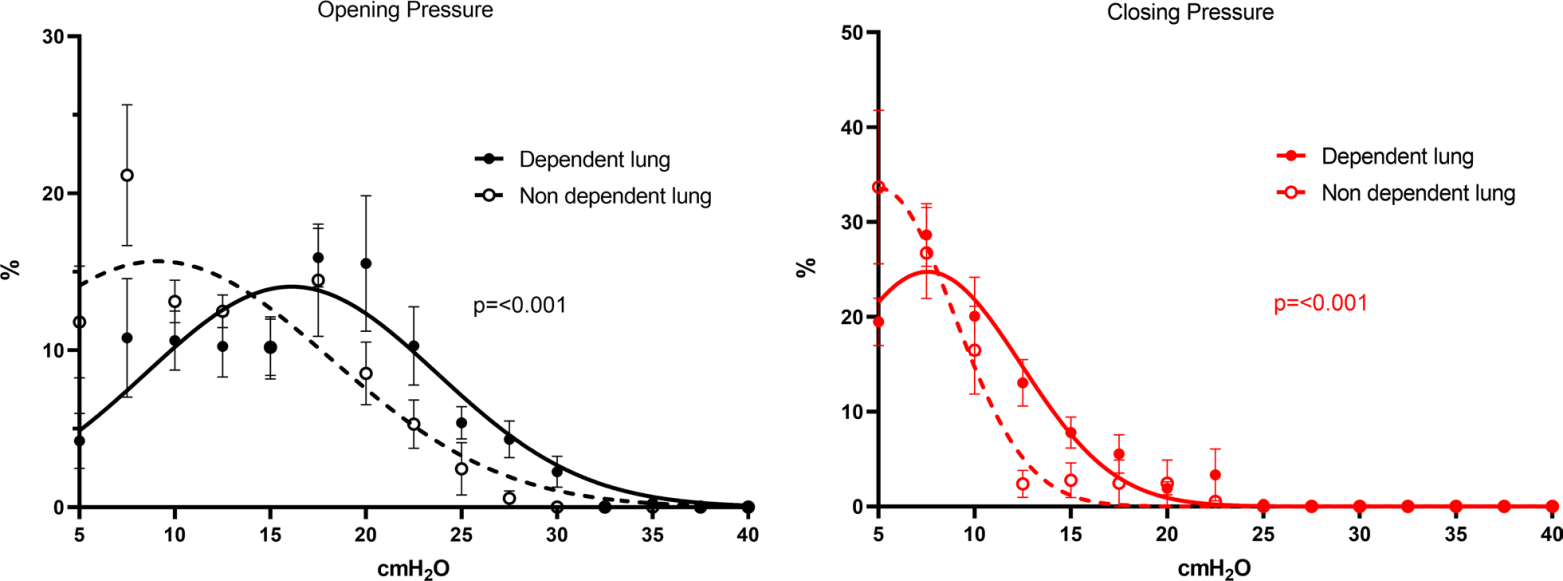
EIT-derived distribution of opening/closing pressures in the dependent and non-dependent lung region. Distribution of opening and closing pressures in the dependent (full line) and non-dependent (dotted line) lung. Mean ± SEM of 8 patients, Gaussian distribution, extra sum of fit, F-Test. Pixels with pressure–volume equation fitting R^2^ < 0.9 were removed from the analysis

When analyzing data from the two ROIs, the Gaussian distribution of OP_pixel_ and CP_pixel_ differed significantly (Fig. 1) both in the ROI_ND_ (mean value of OP_pixel_ 9.1 ± 9.0 cmH_2_O; mean value of CP_pixel_ 5.1 ± 3.8 cmH_2_O) and in the ROI_D_ (mean value of OP_pixel_ 16.1 ± 7.6 cmH_2_O; mean value of CP_pixel_ 7.6 ± 4.8 cmH_2_O). Comparing OP_pixel_ and CP_pixel_ between ROI_D_ and ROI_ND_, we found that their distribution differed significantly, with higher values in the dependent region (Fig. 3).

### Atelectrauma and hysteresis

Median AtI in the whole population was 15.4% (13.1–25.6%) with a maximum of 32.7% and a minimum of 2.3% (Table 2). We disclosed a significant difference in the regional value of AtI, being the latter higher in the dependent lung (p = 0.02, Table 2). Hysteresis measures are reported in Table 2. We found a significant difference in HysMAX and Pao_HysMAX_ between the dependent and non-dependent lung (respectively, p = 0.02 and p = 0.008), being both HysMAX and Pao_HysMAX_ higher in the dependent lung. HysMAX showed a significant correlation with the mean OP_pixel_ both in the global (Rs = 0.76, p = 0.04) and in the dependent lung (Rs = 0.86, p = 0.01). Moreover, HysMAX was significantly correlated with driving pressure (Rs = 0.73, p = 0.048).

**Table 2:** Global and regional EIT-derived Hysteresis ad Atelectrauma index

	Global	Non-dependent	Dependent	p*
HysMAX	0.26 [0.23–0.28]	0.24 [0.23–0.26]	0.30 [0.25–0.39]	0.02
HysPAO (cmH_2_O)	20 [16–22]	16 [15–20]	21 [20–24]	0.008
Atelectrauma index (%)	15.4 [13.1–25.6]	6.6 [2.2–14.1]	38.35 [14.6–52.7]	0.02

Median [IQR] values of global and normalized maximal hysteresis (HysMAX), corresponding pressure location (HysPAO) and atelectrauma index

^*^p = Wilcoxon signed-rank test between the non-dependent and dependent lung region

## Discussion

In the current study, we measured regional opening and closing pressure from pixel-level PV curves obtained by electrical impedance tomography. We described how the pressure determining opening and closing of alveolar units, the intensity of atelectrauma (i.e., the magnitude of atelectrauma index) and the separation between inspiration and expiration due to hysteresis are gravity-dependent, with worse scenario for the dorsal lung. Moreover, all these measures showed large inter-patient variability, indicating the need of bedside monitoring to appreciate the patient’s own regional characteristics.

Ventilator-induced lung injury can worsen ARDS through several mechanisms [19]. In ARDS, lungs are characterized by increased lung weight and surfactant dysfunction, leading to heterogeneous distribution of lung edema and atelectasis [7, 20]. During tidal ventilation, if the opening pressure of a lung unit is reached, the unit will open; during expiration, when the closing pressure is surpassed, the unit will close again. As opening is associated with high stress caused by the passage of the air bubble on the epithelial cells [21], this phenomenon of cyclic opening and closing (atelectrauma) is a main determinant of VILI. This phenomenon of cyclic opening/closing of lung units is heterogeneous. We confirmed that OP_pixel_ and CP_pixel_ have a Gaussian distribution, with higher values for OP. Opening and closing pressures were also higher in the dependent lung, underlying the role of gravity in the distribution of lung edema and transpulmonary pressure [22]. We disclosed values of opening pressure beyond 30 cmH_2_O, confirming that recruitment is a continuous phenomenon [23] during tidal breath insufflation [3, 5].

These findings confirm previous description obtained using the lung CT scan [6] and, more recently, EIT in ALI patients [24]. Moreover, the distribution on OP and CP differed from patient to patient (see Additional file 1: *online supplement*), underlying the need of individualized therapy when applying mechanical ventilation. In this context, this study supports the possibility to assess these phenomena at the bedside, avoiding transport to the radiology department and exposition to ionizing radiation.

Lung hysteresis is a known phenomenon characterized by the presence of a different volume at the same pressure during inspiration and expiration [25]. Several mechanisms have been proposed to justify this behavior, including surfactant effect [8, 26] and stress relaxation. Lung hysteresis indicates that higher energy is required to open the lung that to keep it open and that the extra amount of energy is dissipated between inspiration and expiration into the system [27]. We found that also hysteresis is heterogeneous, with higher values in the dependent lung. This is probably correlated with lower initial alveolar volume and greater volume excursion in the dependent lung [28] where the major part of tidal recruitment is thought to happen and where the atelectrauma index was higher. HysMAX, moreover, showed a good correlation with mean opening pressure. All these data confirm that HysMAX during two low-flow PV maneuvers reflects the extent of alveolar opening and closing and thus the recruitability, as previously found by Demory et al. [29] and suggested by Koefoed-Nielsen et al. [30, 31]. Moreover, we found that this phenomenon happens more in the dependent lung, where the atelectrauma index showed a higher value and hysteresis was higher confirming classical view of where atelectrauma is thought to happen [12].

Minimizing VILI during mechanical ventilation can be crucial to improve the outcome of ARDS. Until now, no available mean exists to detect the risk of atelectrauma in different regions of the lung at the bedside in ARDS, since the pressure–volume curve of the respiratory system can be characterized by overlapping information in such heterogeneous diseases [9]. Positive end-expiratory pressure can counteract the tendency of dorsal lung collapse, but the mechanical information coming from the ventilator (e.g., driving pressure, stress index) contains averaged information from areas with different mechanical behaviors and therefore is not useful to highlight this phenomenon. We showed that by combining pressure/volume curves and EIT it is possible to determine opening/closing pressure at the bedside. Their distribution was highly variable between lung regions and from patient to patient (Additional file 1: figure S2), and therefore, by using EIT, it would be possible to furtherly individualize protective mechanical ventilation to limit regional atelectrauma, instead of using average global indexes like driving pressure.

This technique, applied at the bedside, may increase the pathophysiological information conveyed by EIT. Indeed, by evaluating the percentage of lung units opening and closing one can 1) quantify the maximum risk of exposure of that specific patient to atelectrauma and 2) select a positive end-expiratory pressure that could potentially guarantee recruitment and counteract derecruitment of both the non-dependent and the dependent lung regions.

Our study has several limitations: First, we started the PV maneuver at PEEP = 5 cmH_2_O and not from functional residual capacity; this was done because a reduction in PEEP below 5 cmH_2_O could expose the patients to excessive derecruitment and hypoxemia. Second, we analyzed a relatively small number of patients, none with severe ARDS. These findings must be confirmed therefore in a larger and more severe population. Third, we referred to atelectrauma as the pixels opening/closing between 5 and 40 cmH_2_O, in order to characterize the physiology of each patient. However, atelectrauma is classically defined as intratidal opening/closing of alveolar unit and the intratidal difference in pressure (Pplat/PEEP) is usually lower that the explored one (5–40 cmH_2_O). Forth, no image registration process was used to track the moving parenchyma, as done for the analysis of terminal elements (alveolar units) in CT scan. In EIT imaging, the image is reconstructed in a 2D matrix according to the thorax dimension, and therefore, the pixel dimension varies according to inflation/deflation. This could overcome, at least partially, the problem of image registration seen in fixed pixel-size imaging techniques (e.g., CT scan). Fifth, EIT do not cover the entire lung area but only the tissue around the belt position and the EIT pixel can be characterized by an intrinsic heterogeneity that could not be highlighted by the technique. Finally, the amount of recruitment/derecruitment can be influenced dynamically by time and it could be underestimated by the quasi-static punctual evaluation of the pressure–volume relationship.

## Conclusions

Electrical impedance tomography can highlight regional opening and closing pressures at the bedside in patients affected by ARDS and therefore improve bedside understanding of patient pathophysiology. Opening pressures are higher that closing pressures and gravity impact them, as well as lung hysteresis and atelectrauma: Indeed, the dependent lung is more prone to worse physiological condition. Assessment of regional lung behavior during inspiratory and expiratory PV curves could support clinical stratification of patient severity and guide personalized mechanical ventilation settings.

## Supplementary information

**Additional file 1**. Supplemental tables + patient’s individual regional opening/closing pressures.

## Data Availability

The datasets used and/or analyzed during the current study are available from the corresponding author on reasonable request.

## Abbreviations

ARDS: Acute respiratory distress syndrome
AtI: Atelectrauma index
Ati_D_: Dependent atelectrauma index
AtI_ND_: Non-dependent atelectrauma index
BMI: Body mass index
CP: Closing pressure
CP_pixel_: EIT-derived closing pressure
CT: Computed tomography
Dep: Dependent
EIT: Electrical impedance tomography
f: Flow
HysMax: Normalized maximal hysteresis
IBW: Ideal body weight
LIP: Lower inflection point
LIP_pixel_: Pixel-level lower inflection point
Ndep: Non-dependent
OP: Opening pressure
OP_pixel_: EIT-derived opening pressure
Pao: Airway opening pressure
PaoHysMAX: Pressure corresponding to the point if maximal hysteresis
PEEP: Positive end expiratory pressure
Pplat: Plateau pressure
PV: Pressure–volume
PVpixel: Pixel-level pressure–volume curves
ROI_D_: Dependent lung
ROI_ND_: Non-dependent lung
VILI: Ventilator-induced lung injury
Vt: Tidal volume
