# Supplementary material for: Gravitational distribution of regional opening and closing pressures, hysteresis and atelectrauma in ARDS evaluated by electrical impedance tomography
Source: Crit Care. 2020 Oct 22;24:622. doi: 10.1186/s13054-020-03335-1 (PMC7579854; doi:10.1186/s13054-020-03335-1)
Supplement: Supplementary file 1 — Additional file 1. Supplemental tables + patient’s individual regional opening/closing pressures. [file 13054_2020_3335_MOESM1_ESM.pdf]

Gravitational distribution of regional opening and closing pressures, hysteresis and atelectrauma in ARDS evaluated by Electrical impedance tomography

Gaetano Scaramuzzo, Elena Spinelli, Savino Spadaro, Alessandro Santini, Donatella Tortolani, Francesca Dalla Corte, Antonio Pesenti, Carlo Alberto Volta, Giacomo Grasselli, Tommaso Mauri.

Online data supplement

Table S1: Goodness of fit (Gaussian distribution) in each patient.

	Global		Dependent		Non-dependent	
ID	*Opening pressure*	*Closing pressure*	*Opening pressure*	*Closing pressure*	*Opening pressure*	*Closing pressure*
1	0,68	0,97	0,81	0,98	0,57	0,90
2	0,89	0,83	0,85	0,83	0,92	-
3	0,90	0,96	0,80	0,92	0,87	1,00
4	0,90	0,91	0,89	0,90	0,91	0,92
5	0,85	0,97	0,79	0,95	0,85	0,99
6	0,82	0,98	0,88	0,96	0,92	0,91
7	0,86	0,97	0,90	0,95	0,83	1,00
8	0,83	0,71	0,79	0,46	0,80	0,63

Goodness (R^2) of inflection points gaussian distribution fitting for each patient. Inspiratory and expiratory regional PV curves.

S2: Individual distribution of opening/closing pressures.

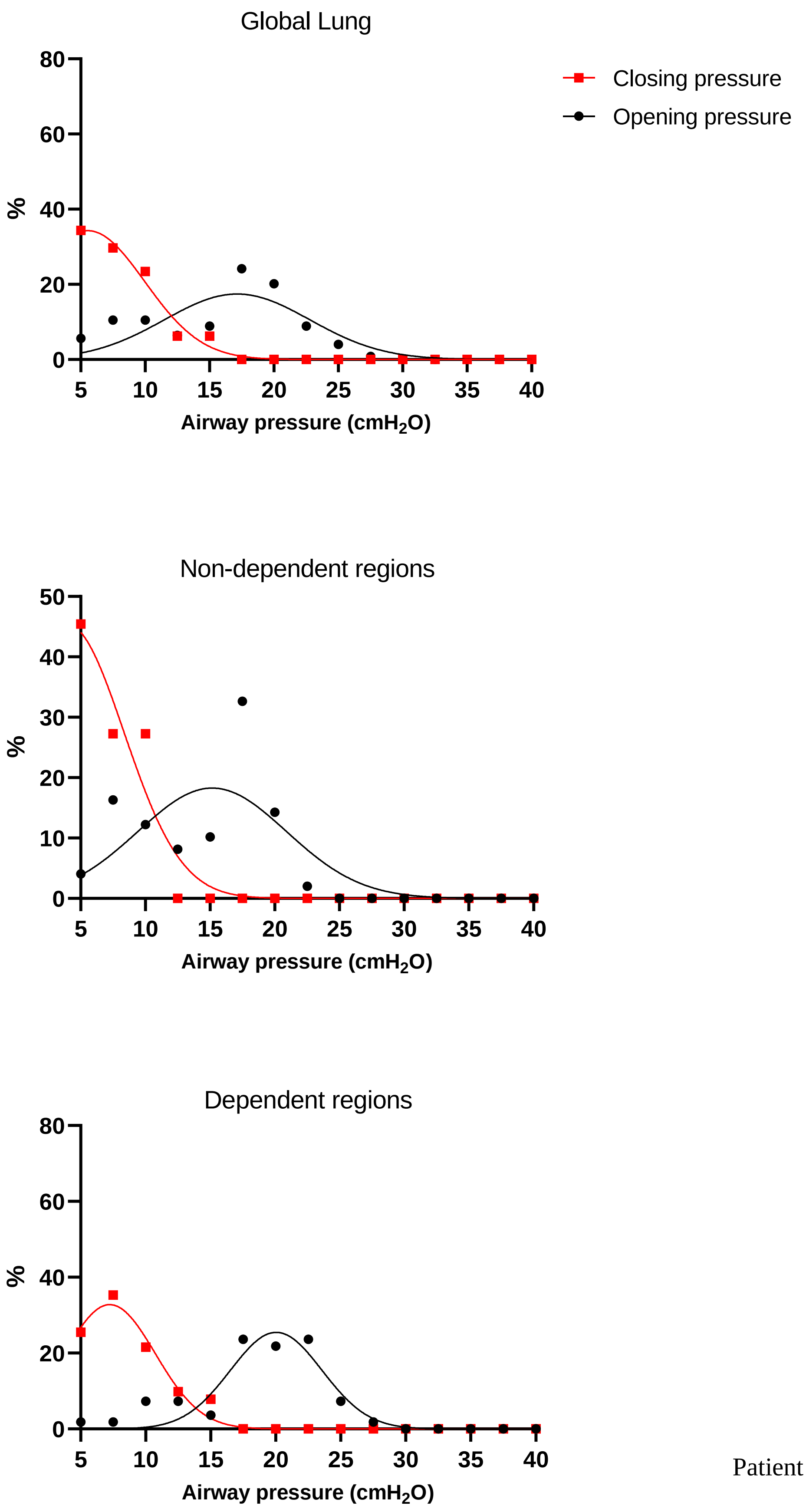

Patient 1

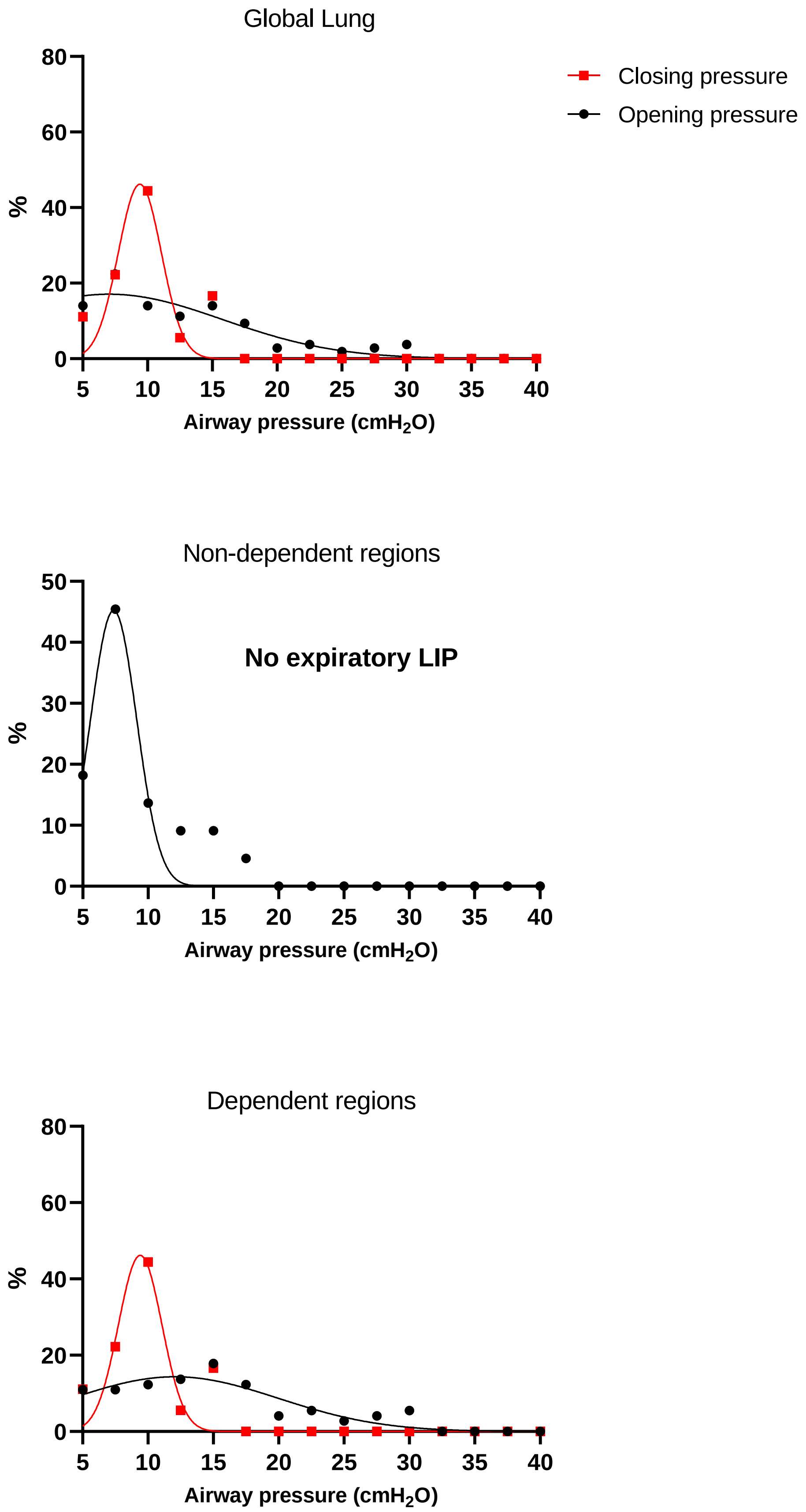
Global Lung
Closing pressure
Opening pressure
%
80
60
40
20
0
5
10
15
20
25
30
35
40
Airway pressure (cmH2O)
Non-dependent regions
No expiratory LIP
50
40
30
20
10
0
Airway pressure (cmH2O)
Dependent regions
Airway pressure (cmH2O)

Patient 2

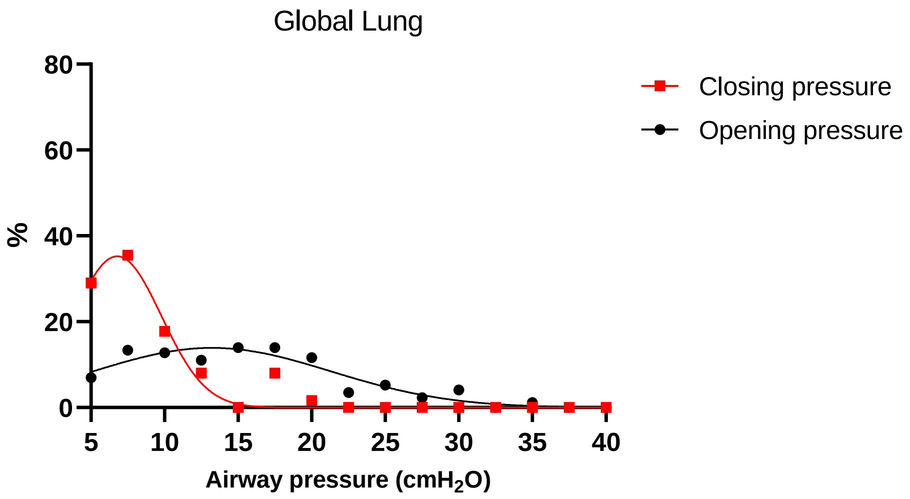
Global Lung
Closing pressure
Opening pressure
%
80
60
40
20
0
5
10
15
20
25
30
35
40
Airway pressure (cmH2O)

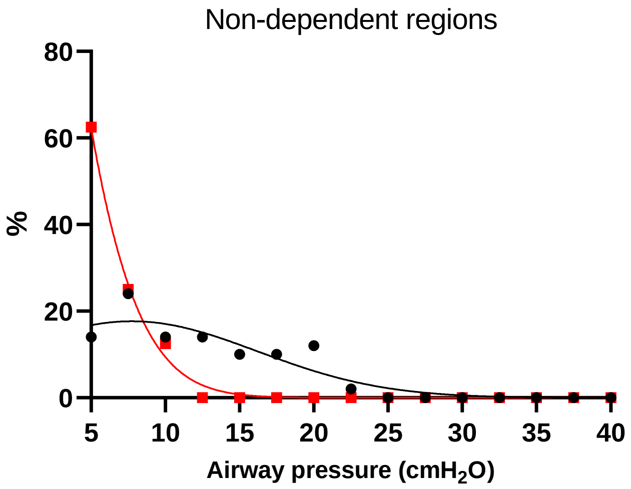
Non-dependent regions
%
80
60
40
20
0
5
10
15
20
25
30
35
40
Airway pressure (cmH2O)

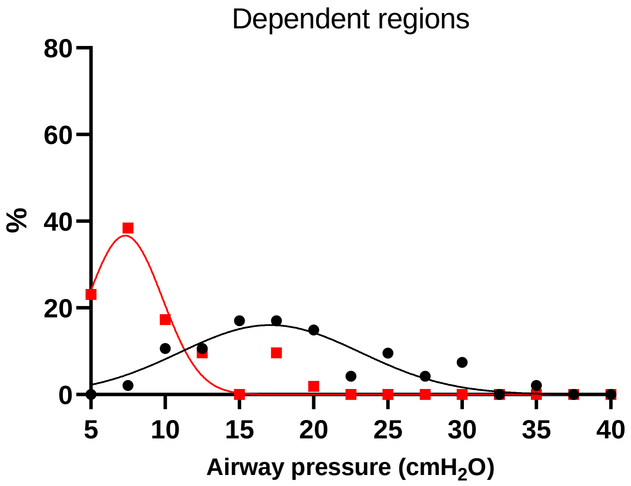
Dependent regions
%
80
60
40
20
0
5
10
15
20
25
30
35
40
Airway pressure (cmH2O)

Patient 3

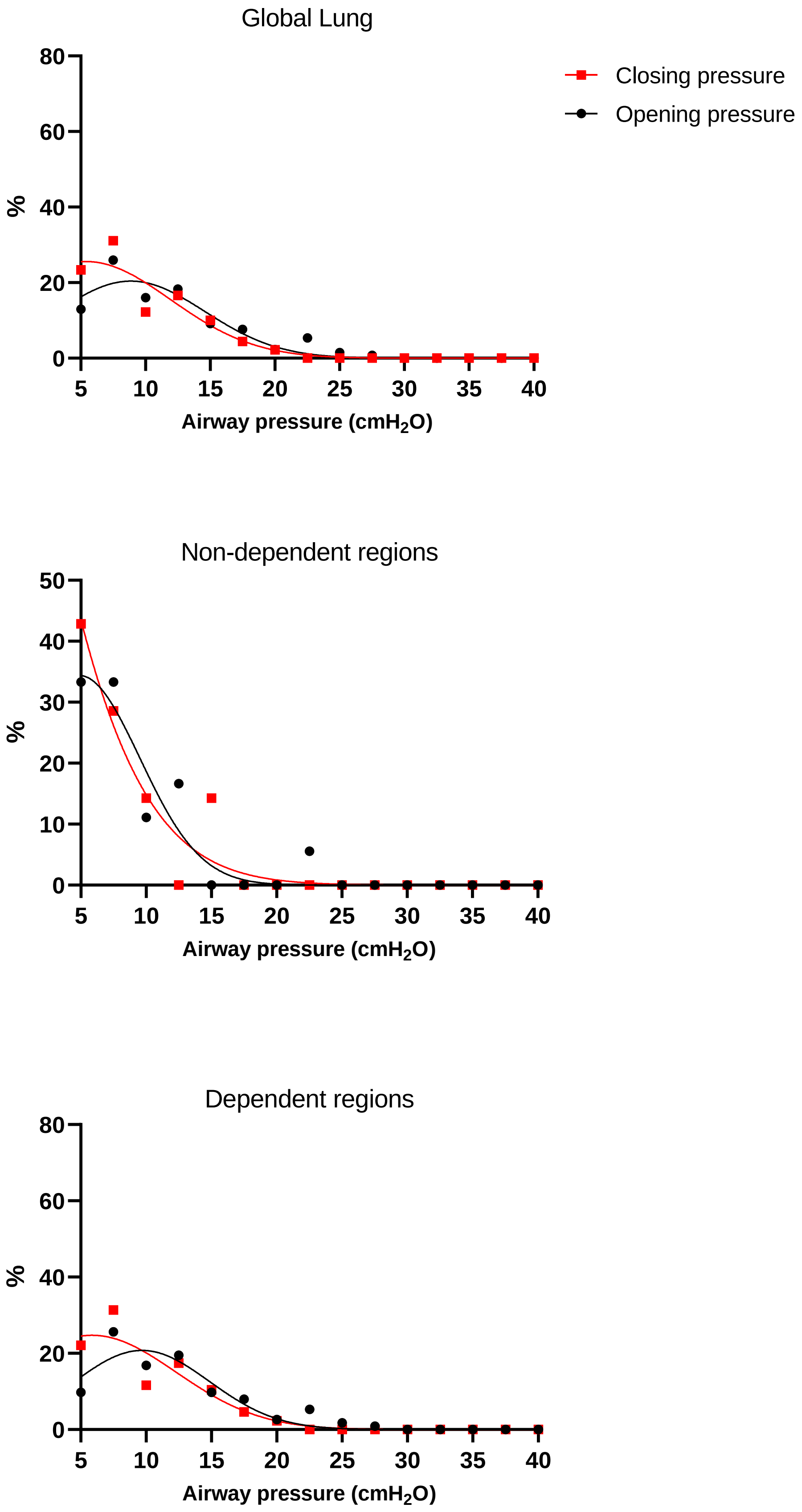
Global Lung
Closing pressure
Opening pressure
%
80
60
40
20
0
5
10
15
20
25
30
35
40
Airway pressure (cmH2O)
Non-dependent regions
%
50
40
30
20
10
0
5
10
15
20
25
30
35
40
Airway pressure (cmH2O)
Dependent regions
%
80
60
40
20
0
5
10
15
20
25
30
35
40
Airway pressure (cmH2O)

Patient 4

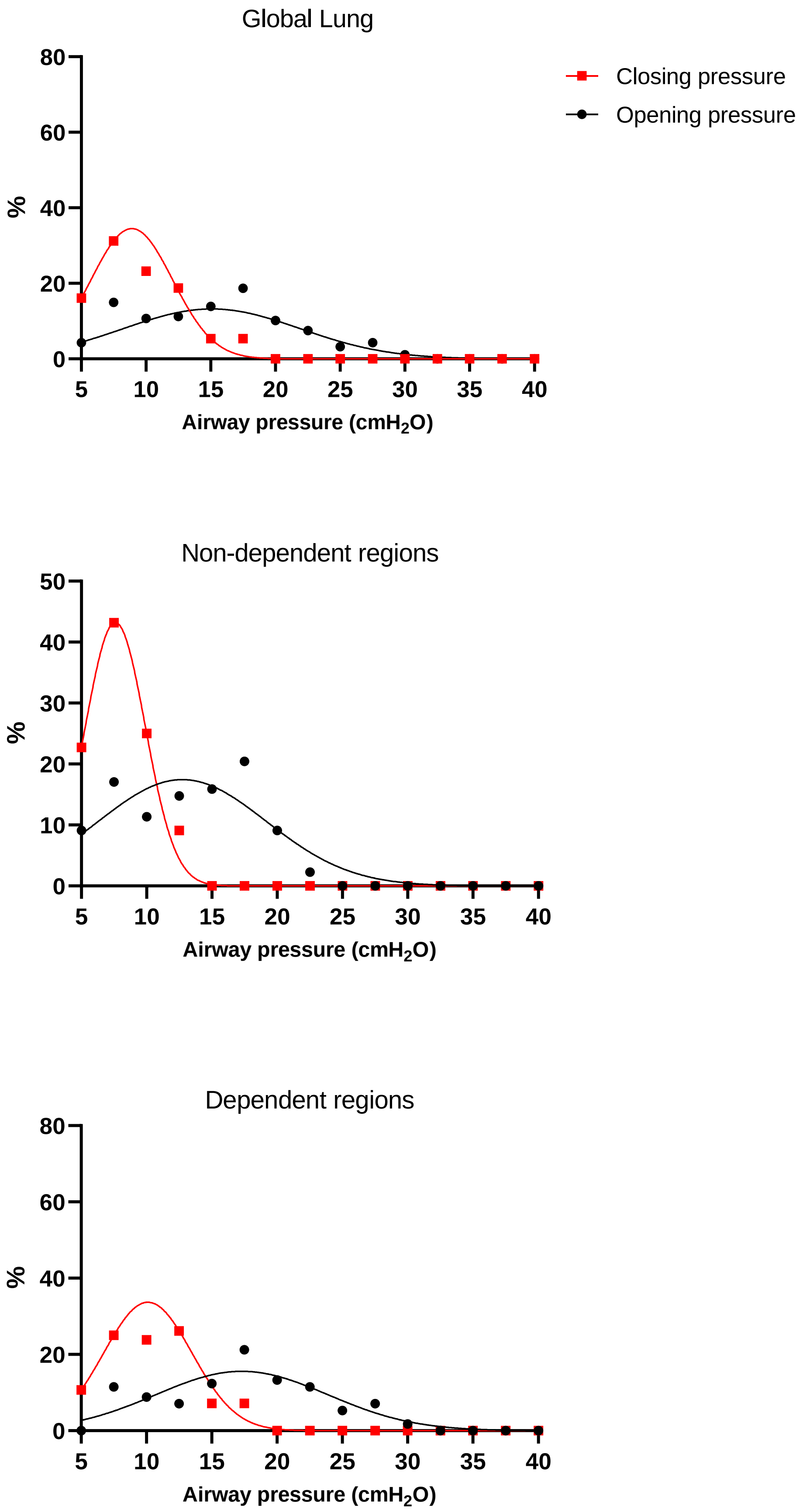
Global Lung
Closing pressure
Opening pressure
%
80
60
40
20
0
5 10 15 20 25 30 35 40
Airway pressure (cmH2O)
Non-dependent regions
%
50
40
30
20
10
0
5 10 15 20 25 30 35 40
Airway pressure (cmH2O)
Dependent regions
%
80
60
40
20
0
5 10 15 20 25 30 35 40
Airway pressure (cmH2O)

Patient 5

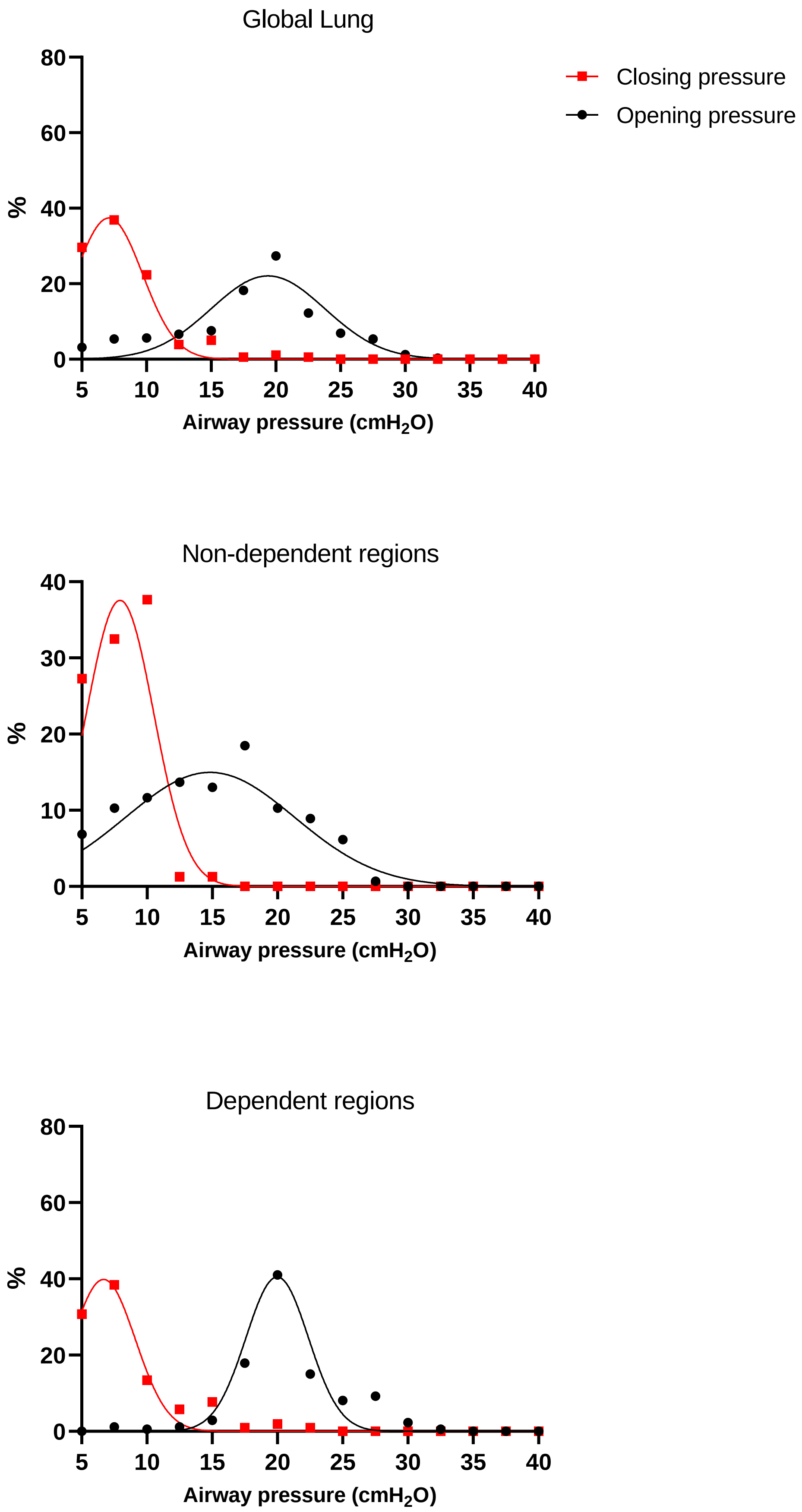
Global Lung
Closing pressure
Opening pressure
%
80
60
40
20
0
5
10
15
20
25
30
35
40
Airway pressure (cmH2O)
Non-dependent regions
40
30
20
10
0
Dependent regions

Patient 6

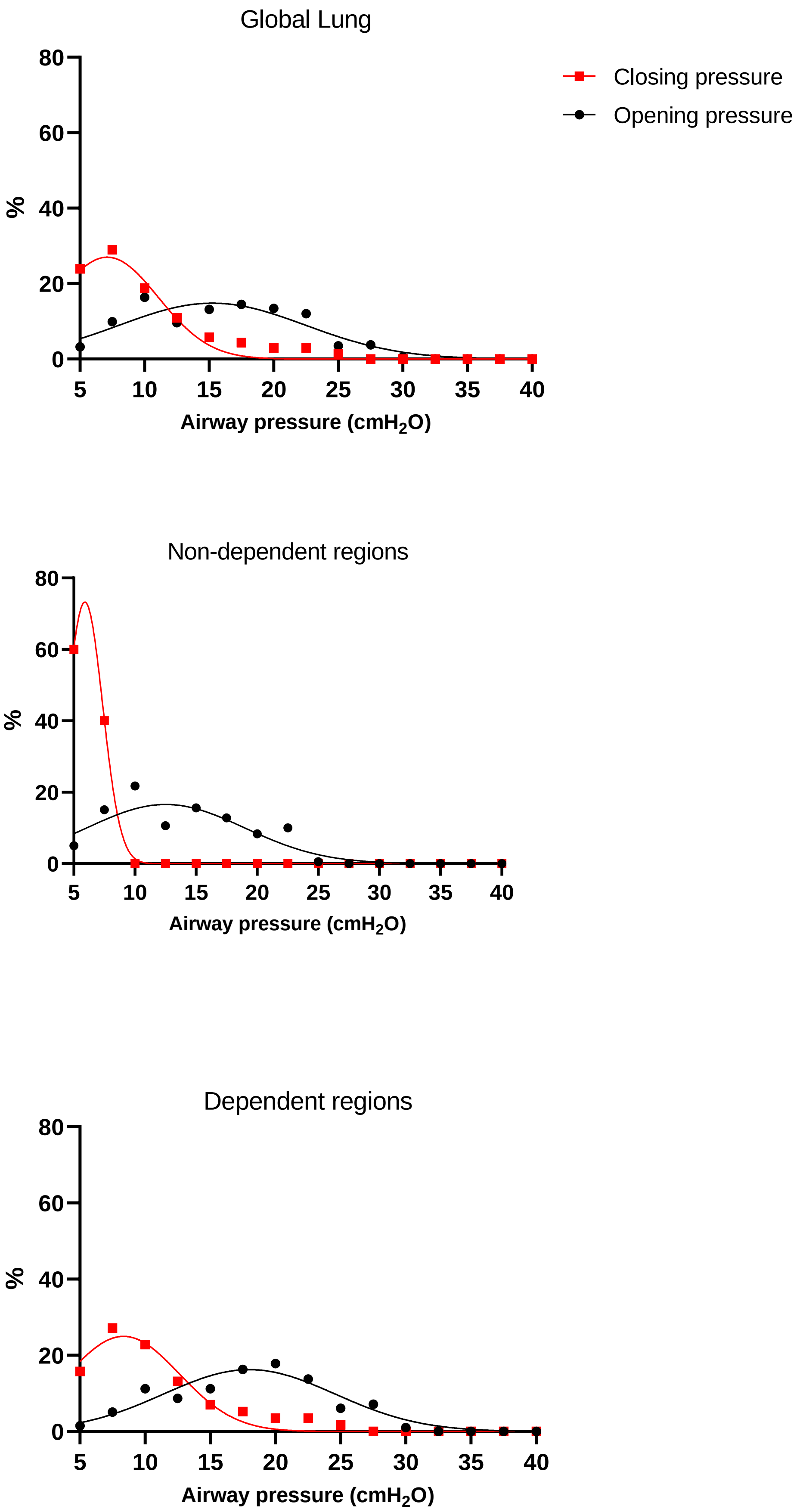
Global Lung
Closing pressure
Opening pressure
%
80
60
40
20
0
5
10
15
20
25
30
35
40
Airway pressure (cmH_2O)
Non-dependent regions
%
80
60
40
20
0
5
10
15
20
25
30
35
40
Airway pressure (cmH_2O)
Dependent regions
%
80
60
40
20
0
5
10
15
20
25
30
35
40
Airway pressure (cmH_2O)

Patient 7

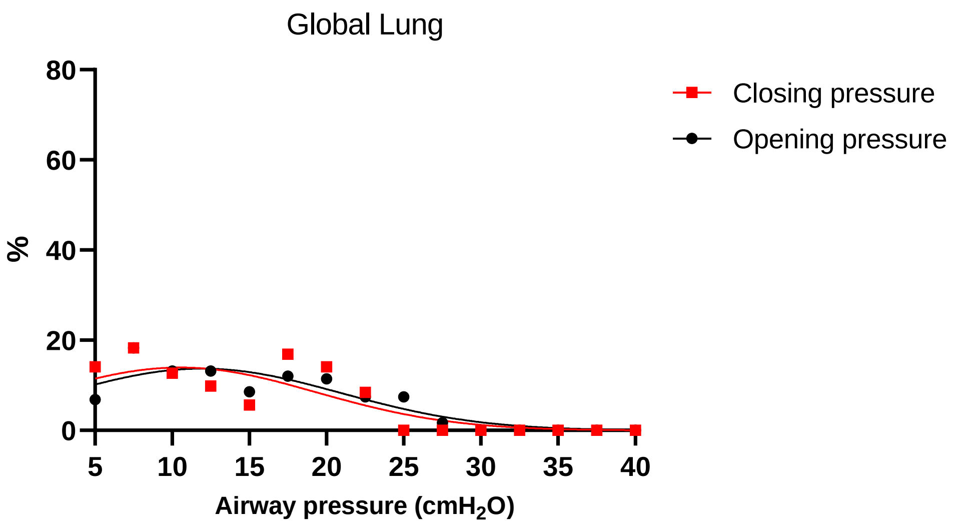

Global Lung
Closing pressure
Opening pressure
80
60
40
20
0
%
5
10
15
20
25
30
35
40
Airway pressure (cmH$_2$O)

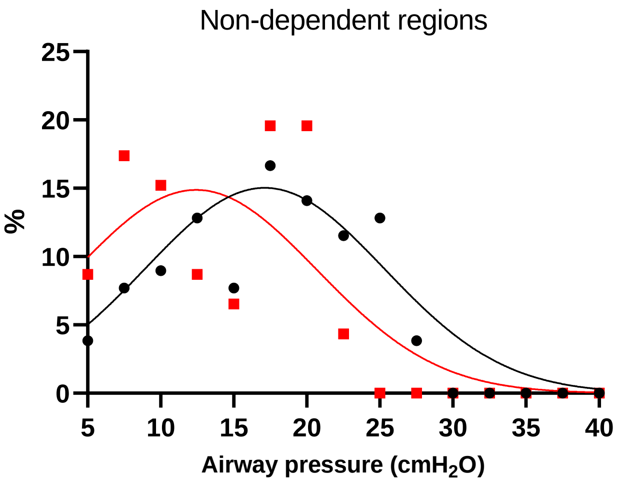

Non-dependent regions
25
20
15
10
5
0
%
5
10
15
20
25
30
35
40
Airway pressure (cmH$_2$O)

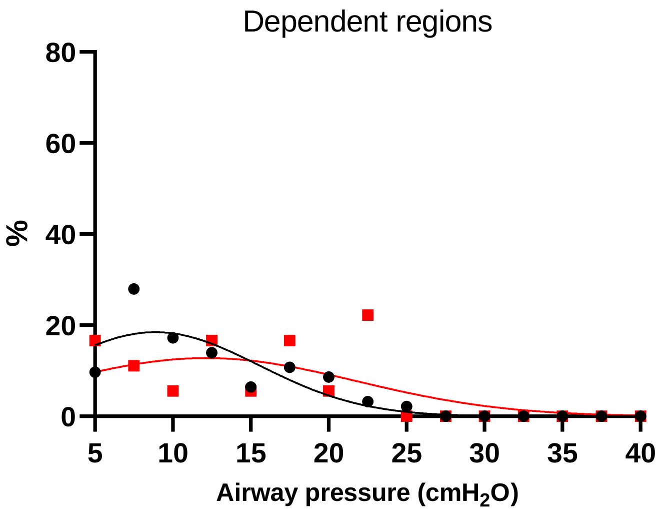

Dependent regions
80
60
40
20
0
%
5
10
15
20
25
30
35
40
Airway pressure (cmH$_2$O)

Patient 8

S3: Number of ventilated pixels, LIP and bad fitting in the inspiratory and expiratory regional PV curves. Individual and global results.

	**Inspiratory PV**			**Expiratory PV**		
Patient	*Ventilated pixels*	*Number of LIP*	*Bad fitting*	*Ventilated pixels*	*Number of LIP*	*Bad fitting*
1	339	142	0	346	145	2
2	522	220	0	529	44	0
3	391	206	0	393	147	0
4	542	241	0	543	217	0
5	439	248	2	465	160	3
6	489	352	0	498	310	0
7	507	418	3	485	288	0
8	400	222	6	398	107	2
Total	3629	2049 (56.5%)	11 (0.3%)	3657	1418 (38.8%)	7 (0.2%)

S4: HysMAX and PaoHysMAX calculation in a representative patient.

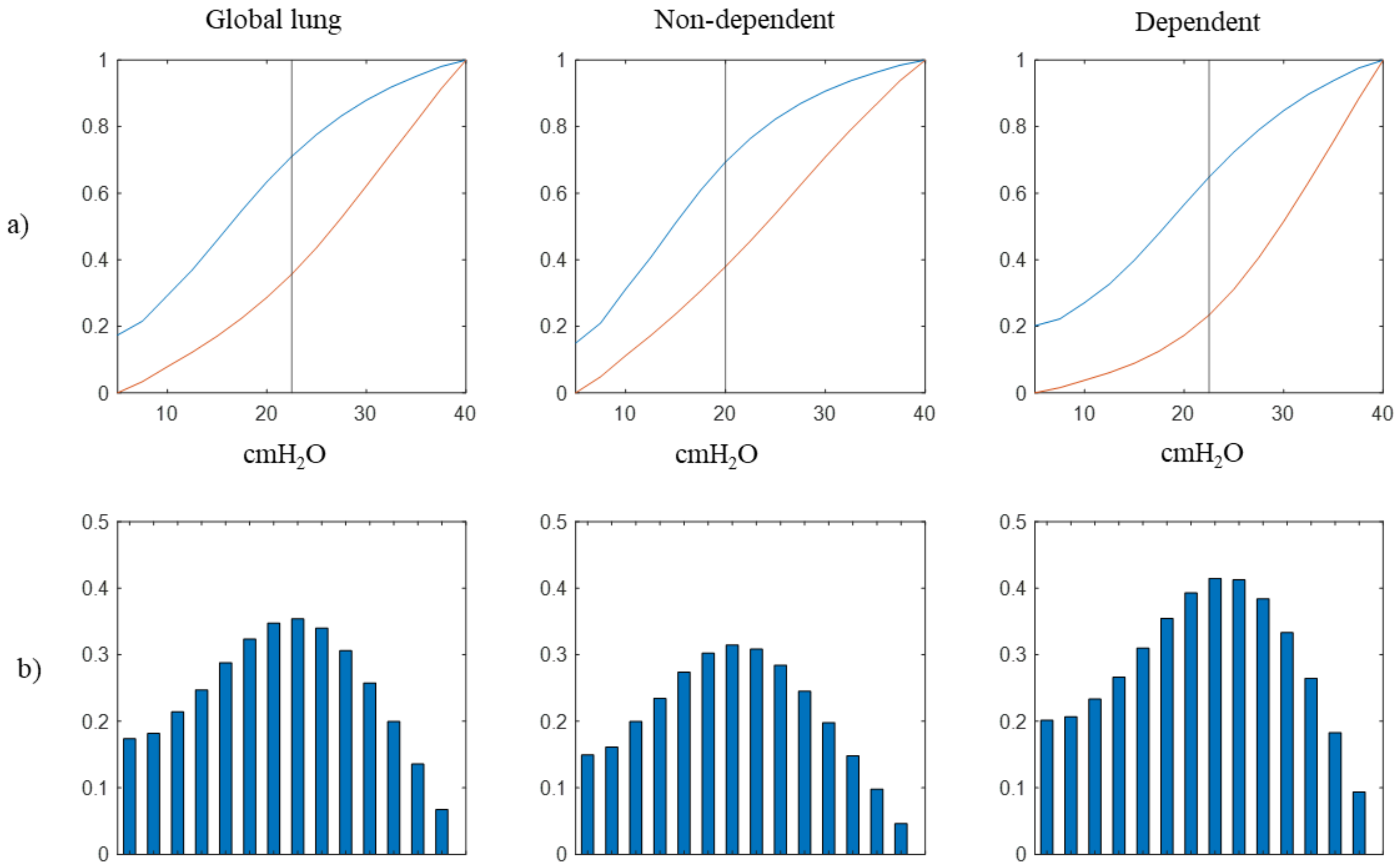

a) Inspiratory (red) and expiratory (blue) pressure-volume curves for the global, dependent and non-dependent lung. Each curve was normalized between 0 and 1. b) difference, at each pressure interval of 2.5 cmH_2O, between the expiratory and inspiratory limb of the PV curve of the corresponding curves. The vertical line in a) represents the level of HysMAX and PaoHysMAX.

Table S5: Opening/closing pressure distribution comparison

	Opening pressure			*Closing pressure*				
	AREA	*SD*	*Mean*	*AREA*	*SD*	*Mean*	*p**	*F(DFn, DFd)*
Global	293	8	13.5	332.5	5.1	6.8	<0.0001	24.75 (3, 24)
Dependent	268,5	7.6	16.1	300.4	4.8	7.6	<0.0001	44.66 (3, 24)
Non-dep	352,9	9	9.1	321.4	3.8	5.1	<0.0001	34.19 (3, 24)
	Dependent			*Non-dependent*			*p**	*F(DFn, DFd)*
Opening	268.5	7.6	16.1	352.9	9	9.1	0.0008	7.930 (3, 24)
Closing	300.4	4.8	7.6	321.4	3.8	5.1	<0.0001	20.13 (3, 24)

Comparison of gaussian distributions between opening/closing pressures and between dependent/non-dependent lung regions. Extra sum of squares, F test.

Figure S6: Comparison of regional compliance between the non-dependent and the dependent lung region

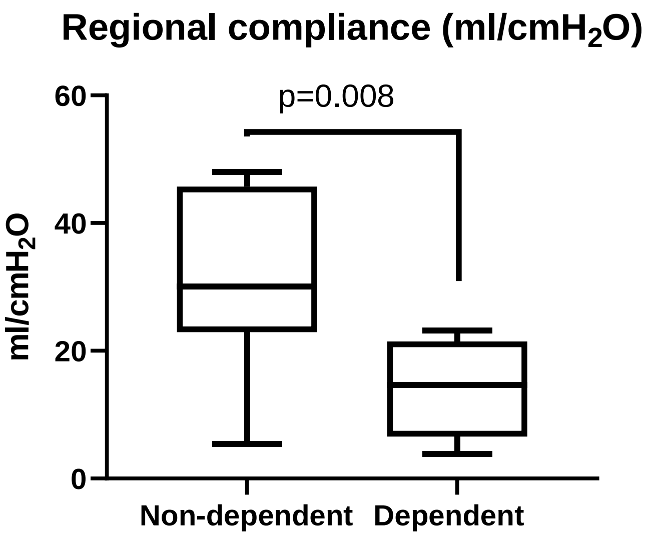

Regional compliances were calculated using the relative variation of volume in each ROI during the PV maneuver on the variation of pressure (35 cmH_2O) used to perform the maneuver.
